# Functional magnetic resonance imaging for clinical evaluation of uterine contractility

**DOI:** 10.1590/S1679-45082018MD3863

**Published:** 2018-04-06

**Authors:** Vinicius Adami Vayego Fornazari, Stela Adami Vayego, Denis Szejnfeld, Jacob Szejnfeld, Suzan Menasce Goldman

**Affiliations:** 1 Universidade Federal de São Paulo, São Paulo, SP, Brazil; 2 Universidade Federal do Paraná, Curitiba, PR, Brazil

**Keywords:** Uterine contraction, Myoma, Magnetic resonance imaging, Infertility, Sperm transport, Contração uterina, Mioma, Imagem por ressonância magnética, Infertilidade, Transporte espermático

## Abstract

Uterine contractility out of the gestational phase, during the menstrual cycle and the habitual functional variations of the organ, this is one of the responsible mechanisms for reproduction and fertility, due to its direct action in the mechanisms conducting the spermatozoa to the ovule and in the decidual implantation. Pathologies such as uterine leiomyoma, endometriosis, adenomyosis, polycystic ovarian syndrome, as well as the use of intrauterine devices and oral contraceptives, may alter a functionality of uterine contractility. Thus, magnetic resonance imaging with ultrafast sequences provides a dynamic evaluation (cine-MRI) and thus the correlation of uterine contractility quality in patients with current infertility or pathologies.

## RESUMO

A contratilidade uterina fora da fase gestacional, durante o ciclo menstrual e as habituais variações funcionais do órgão, é um dos mecanismos responsáveis pela reprodução e fertilidade, devido sua ação direta nos mecanismos de condução dos espermatozoides até o óvulo e na implantação decidual. Patologias como leiomioma uterino, endometriose, adenomiose, síndrome dos ovários policísticos, bem como o uso de dispositivos intrauterinos e anticoncepcionais orais, podem alterar a funcionalidade da contratilidade uterina. Desta forma a ressonância magnética com sequências ultra-rápidas proporcionam uma avaliação dinâmica (cine-RM) e assim a correlação da qualidade da contratilidade uterina em pacientes com infertilidade ou patologias vigentes.

## INTRODUCTION

**Figure 1:**
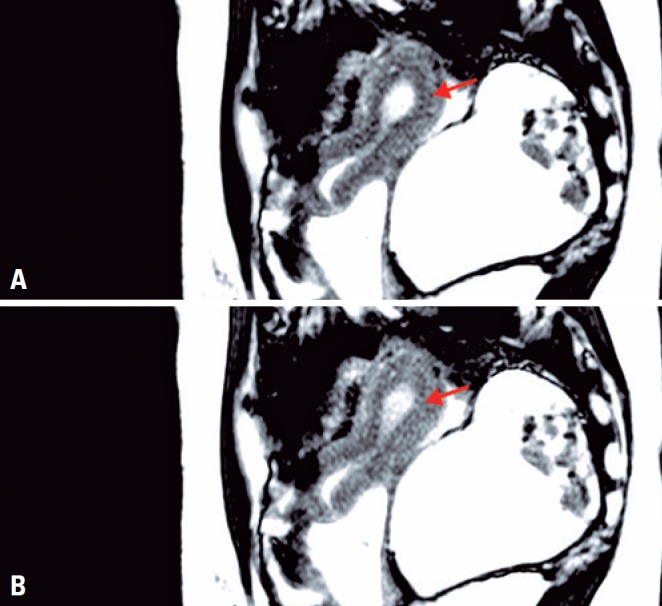
Uterine pelvis, cine-RM sequence on sagittal plane, with focal contraction uterine fundus. (A) Uterus without contraction, note homogeneous, regular junctional zone in the posterior aspect of the fundus. (B) Uterus with contraction, note imprint/narrowing of the junctional zone in the posterior aspect of the fundus, as it becomes thinner in response to uterine peristalsis

Uterine function is intuitively associated with the gestational period. However, the non-pregnant uterus has other vital functions, such as elimination of endometrial cells during the menstrual period and promotion of sperm cell transport to implantation sites, both related to myometrial peristalsis (Figures 1A and 1B).^(^ ^1^ ^,^ ^2^ ^)^

**Figure 2:**
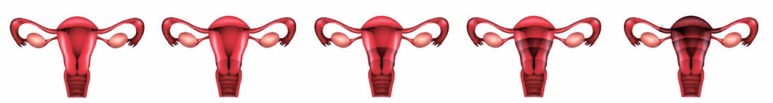
Illustration of wave dynamics in type A cervico-fundal contractions

**Figure 3:**
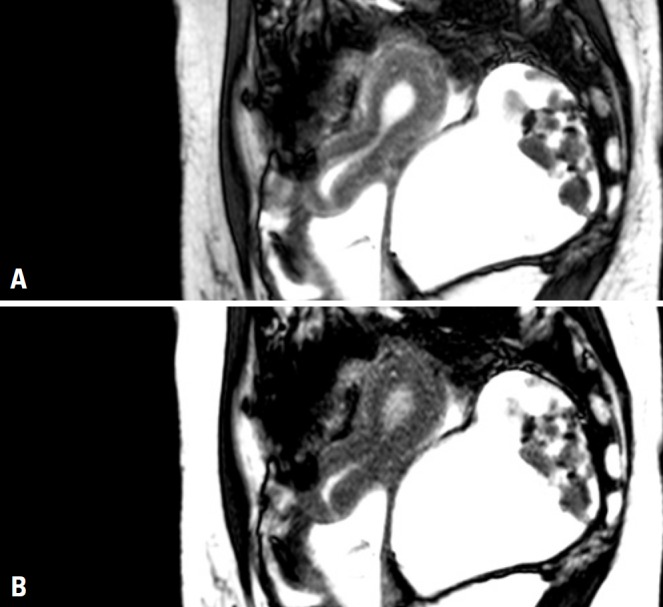
Uterine pelvis, cine-RM sequence on sagittal plane, with focal contraction uterine cervix. (A) Uterus without contraction. (B) Uterus with contraction, note the focal uterine contraction at cervix level, with projection of the endometrial canal and mild thinning of the junctional zone

**Figure 4:**
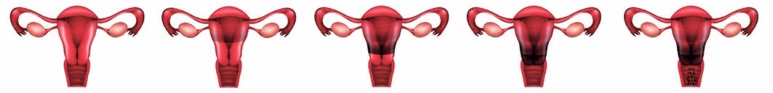
Illustration of wave dynamics in type B fundo-cervical contractions

Three types of uterine contractions (or peristalsis) may be distinguished in women, which vary in intensity and frequency according to the functional and circadian rhythm.^(^ ^1^ ^,^ ^2^ ^)^ For educational purposes, these may be divided as follows: Type A (cervico-fundal peristalsis) – prevailing in the follicular and luteal phases, with increased intensity and frequency in the preovulatory phase (Figures 2, 3A and 3B);^(^ ^1^ ^,^ ^2^ ^)^ type B (fundo-cervical peristalsis) – decreasing frequency and intensity, disappearing at mid-cycle (Figure 4);^(^ ^1^ ^,^ ^2^ ^)^ and type C – incomplete peristaltic contractions arising from the isthmus towards the lower central portion of the uterine body.^(^ ^1^ ^,^ ^2^ ^)^ Type A and C contractions occur in the luteal phase, with decreasing frequency towards the end of the cycle. As a result, approximately one complete cervicofundal contraction occurs per second in the mid-luteal phase, regressing to one-fourth at the end of this phase and revealing a quiescent period of the uterine fundus.^(^ ^2^ ^)^ Different types of contractions support the hypothesis that uterine peristalsis is associated with the initial reproductive stages, *i.e.*, sperm cell transport in the preovulatory phase and preservation of incipient pregnancy.^(^ ^1^ ^)^

Uterine contractility is not constant over the course of the reproductive phase. Rather, it varies according to the menstrual cycle and is negatively affected by pathological conditions, such as uterine leiomyoma, endometriosis, adenomyosis and polycystic ovary syndrome, or even by intrauterine (IUD) and oral (OCP) contraceptives.^(^ ^1^ ^-^ ^9^ ^)^

In a study evaluating 12 women free from pelvic conditions during the preovulatory phase, Kido et al., failed to detect significant variations in uterine peristalsis during daytime, probably due to the lack of variation in serum ovarian hormone levels (estradiol, luteinizing hormone − LH and follicle-stimulating hormone − FSH).^(^ ^1^ ^)^

The menstrual period is responsible for one of the most common uterine conditions affecting young women: primary dysmenorrhea. Increased myometrial prostaglandin production during this period leads to increased uterine contraction frequency and amplitude, reducing uterine blood flow and ultimately causing anoxic pain. The use of functional resonance imaging to investigate dysmenorrhea was proposed by Kataoka et al.^(^ ^3^ ^)^

Endometriosis, a major cause of female infertility, is characterized by the presence of stroma and endometrial glands on the outer surface of the myometrium, within the myometrium (adenomyosis), or even in the ovaries and peritoneum. In a cine-MRI study evaluating women suffering from endometriosis, Kido et al., observed significant decrease in uterine peristalsis occurrence and frequency during the periovulatory phase.^(^ ^4^ ^)^

Leiomyomas are the most common form of gynecological cancer. For this reason, Orisaka et al., investigated the potential impacts of this tumor on uterine peristalsis and concluded that affected women had abnormal peristaltic patterns during the menstrual and luteal phases, leading to prolonged menses and hypermenorrhea, inefficient embryo implantation and increased risk of abortion.^(^ ^5^ ^)^

Intrauterine device has been around for more than two centuries and is an effective contraceptive method; however, its exact mode of action has not been fully understood and is thought to reflect negative impacts on the implantation of the fertilized egg, along with potential spermicidal effects.^(^ ^6^ ^)^ Kido et al., used cine-MRI to investigate IUD users during the periovulatory phase and detected suppression of cervico-fundal peristalsis, reversal to the fundocervical direction and thickening of the junctional zone and endometrium, findings that suggest underlying effects of IUDs on conception.^(^ ^6^ ^)^

Oral contraceptives are also effective and have been in use for more than 30 years. These act primarily by inhibiting ovulation via suppression of basal FSH and LH production in response to gonadotrophin releasing hormone (GnRH), with resulting suppression of estrogen and progesterone peak levels. Other potentially relevant modes of action remain to be confirmed, such as changes in tubal and uterine motility, endometrial maturation and cervical mucus production. In an effort to clarify these issues, Kido et al., evaluated fertile OCP users of reproductive age using cine-MRI and demonstrated dramatic decreases in uterine contractility intensity and frequency (down to 8.6%) in this group as compared to the Control Group (100%). Mid-cycle LH, FSH and estrogen peak suppression suggests major contraction stimulating effects of follicular estrogen. Hence, peristalsis suppression may be considered an adverse effect of OCPs.^(^ ^7^ ^)^

Polycystic ovary syndrome is the most common endocrine metabolic disorder affecting women of reproductive age, and causes infertility due to oligoanovulation. Uterine morphology and function in affected women are poorly understood. According to Leonhardt et al., lack of peristalsis is more prevalent in these patients compared to controls and is thought to be associated with endometrial thickening, with no changes in myometrial morphology.^(^ ^8^ ^)^

### Cine-MRI of the pelvis - assessment of uterine peristalsis and technical description of image acquisition

Up to the 1990s, functional assessment of the uterus in women suffering dysmenorrhea involved the use of electrodes or intrauterine catheters. These devices were capable of measuring muscle tone intensity and the amplitude of uterine contractions, but provided limited morphological detail of the uterus.^(^ ^3^ ^,^ ^10^ ^)^

In 1996 and 2000, Kunz et al., described the use of transvaginal videosonography (TVVS) to assess uterine peristalsis and categorized sound waves according to their respective directions, as follows: A, cervicofundal; B, fundo-cervical; and C, isthmic.^(^ ^2^ ^)^ Radiology was going through a major global revolution at that time, with the advent and rapid growth of magnetic resonance imaging, particularly for pelvic assessment. Then, in 2004 researchers from the University of Kyoto observed that cine-MRI was able to provide more accurate images of peristaltic movements associated with the endometrium, junctional zone and myometrium.^(^ ^9^ ^,^ ^10^ ^)^ The value of cine-MRI in the assessment of pelvic floor weakness associated with cystocele, enterocele, rectocele and uterine or vaginal prolapses should also be emphasized.^(^ ^10^ ^)^

Using visual concepts applied in Television and Cinema, cine-MRI consists of the acquisition of multiple images in a short period of time (seconds) for future, continuous visualization at lower rates, consistent with the visual ability of the human eye (approximately 12 frames/second).^(^ ^10^ ^)^

Cine-MRI has two major applications based on T2 sequences: single shot fast spin echo (SSFSE) and steady-state free precession (SSFP). Despite superior resolution, SSFP generates a lot of noise and is more sensitive to susceptibility and distortion artifacts. For these reasons, along with superior contrast and spatial resolution, SSFSE (Table 1) is the sequence of choice for uterine peristalsis assessment.^(^ ^9^ ^,^ ^10^ ^)^

**Table 1:** Protocol for single shot fast spin echo image acquisition in the absence of cholinergic effects and during quiet breathing. Images of the uterine body acquired in a sagittal plane for 3 minutes (60 images in total). Dynamic visual assessment in cine mode should be approximately 12 or more frames for second

FOV	300 millimeters
TR	3,000 milliseconds
TE	80 milliseconds
Interpolated reconstruction matrix	256×192
	512×384
Time	3 minutes
Slice thickness	10 millimeters
Flip angle	80°
Nex	1

Cine-MRI: magnetic resonance imaging, ultrafast sequences with short time.

Therefore, when used to assess uterine contractility in infertile women suffering from associated pelvic conditions, cine-MRI may contribute additional pathophysiological information, with no significant time and financial constraints.
